# Outcomes Following Abiraterone versus Enzalutamide for Prostate Cancer: A Scoping Review

**DOI:** 10.3390/cancers14153773

**Published:** 2022-08-03

**Authors:** Yash B. Shah, Amy L. Shaver, Jacob Beiriger, Sagar Mehta, Nikita Nikita, William Kevin Kelly, Stephen J. Freedland, Grace Lu-Yao

**Affiliations:** 1 Sidney Kimmel Medical College, Thomas Jefferson University, Philadelphia, PA 19107, USA; yxs049@students.jefferson.edu (Y.B.S.); jacob.beiriger@students.jefferson.edu (J.B.); sagar.mehta@students.jefferson.edu (S.M.); 2 Department of Medical Oncology, Sidney Kimmel Cancer Center, Thomas Jefferson University, Philadelphia, PA 19107, USA; amy.shaver@jefferson.edu (A.L.S.); fnu.nikita@jefferson.edu (N.N.); william.kelly@jefferson.edu (W.K.K.); 3 Department of Surgery, Cedars-Sinai Medical Center, Los Angeles, CA 90048, USA; stephen.freedland@cshs.org; 4 Section of Urology, Durham VA Medical Center, Durham, NC 27705, USA; 5 Jefferson College of Population Health, Thomas Jefferson University, Philadelphia, PA 19107, USA

**Keywords:** androgen deprivation, novel hormonal therapy, urologic oncology, treatment choice, healthcare resource utilization, cancer outcomes, prostate cancer, abiraterone acetate, enzalutamide

## Abstract

**Simple Summary:**

Abiraterone acetate and enzalutamide are novel therapies used in advanced prostate cancer. However, their outcomes and toxicities may differ based on patient–specific factors. Understanding these differences may allow clinicians to make personalized treatment decisions based on individual patients. Because clinical trials do not represent the real-world population, this study used a formal protocol to review and collate smaller, real-world samples, aiming to explain the differences in outcomes following administration of these two drugs. We found that enzalutamide typically has improved cancer response and overall survival. Enzalutamide more commonly causes neurological side effects and fatigue, while abiraterone acetate has cardiovascular complications. Abiraterone acetate leads to increased costs and healthcare needs, including hospitalizations and emergency room visits. Ultimately, this study demonstrates significant differences in patient experiences and outcomes following abiraterone acetate versus enzalutamide. Clinicians may use this information to inform their treatment choice on a patient-specific basis.

**Abstract:**

Abiraterone acetate (AA) and enzalutamide (ENZ) are commonly used for metastatic prostate cancer. It is unclear how their outcomes and toxicities vary with patient-specific factors because clinical trials typically exclude patients with significant comorbidities. This study aims to fill this knowledge gap and facilitate informed treatment decision making. A registered protocol utilizing PRISMA scoping review methodology was utilized to identify real-world studies. Of 433 non-duplicated publications, 23 were selected by three independent reviewers. ENZ offered a faster and more frequent biochemical response (30–50% vs. 70–75%), slowed progression (HR 0.66; 95% CI 0.50–0.88), and improved overall survival versus AA. ENZ was associated with more fatigue and neurological adverse effects. Conversely, AA increased risk of cardiovascular- (HR 1.82; 95% CI 1.09–3.05) and heart failure-related (HR 2.88; 95% CI 1.09–7.63) hospitalizations. Ultimately, AA was associated with increased length of hospital stay, emergency department visits, and hospitalizations (HR 1.26; 95% CI 1.04–1.53). Accordingly, total costs were higher for AA, although pharmacy costs alone were higher for ENZ. Existing data suggest that AA and ENZ have important differences in outcomes including toxicities, response, disease progression, and survival. Additionally, adherence, healthcare utilization, and costs differ. Further investigation is warranted to inform treatment decisions which optimize patient outcomes.

## 1. Introduction

Prostate cancer (PCa) is the most commonly diagnosed non-skin cancer in men, accounting for over 20% of new cancer cases [1,2,3,4]. Abiraterone acetate (AA), an androgen biosynthesis inhibitor, and enzalutamide (ENZ), an androgen receptor signaling inhibitor, are novel hormonal therapies (NHTs) that are mainstay additions to androgen deprivation therapy in PCa, particularly in metastatic disease [5,6,7,8,9,10]. Although the survival rate for locoregional disease approaches 99%, that of advanced and metastatic cancers is markedly lower, making NHT optimization crucial to urologic oncology [1]. There are currently no clinical guidelines for choosing AA versus ENZ, and both are approved for use in largely similar conditions.

Unfortunately, NHT clinical trials frequently exclude patients with significant comorbidities, restricting the generalizability of their findings to the broader population [11]. By understanding real-world outcomes based on disease-specific measures, drug-associated toxicities, patient comorbidities, and broader systemic factors, clinicians can perform an informed risk assessment to guide treatment choice and optimize the patient experience. Notably, AA and ENZ have primarily been compared in small, retrospective cohorts, which are limited in interpretation but offer real-world data, the compilation of which may offer great clinical insight [2].

No adequately powered comparative studies have yet elucidated the real-world risks and benefits of AA versus ENZ. Understanding potential differences in patient survival and cancer response rates may improve PCa outcomes. Furthermore, understanding factors such as patient adherence, healthcare resource utilization (HRU), and total costs can facilitate quality improvement. Given the availability of alternative treatment options, tailored therapy has the potential to improve treatment outcomes.

Importantly, adverse drug effects (ADEs) are common with NHTs and may vary with patient and drug characteristics [8]. Limited real-world studies have shown associations between NHT toxicities and pre-existing metabolic, cardiovascular, or neurological conditions [12,13]. For instance, AA has been shown to significantly increase cardiomyopathy while ENZ increases hypertension [11,13,14,15,16,17,18,19]. Notably, cardiovascular disease (CVD) is the most common comorbidity and cause of death in men with PCa, and CVD incidence is higher in men with PCa compared to the general population, making treatment evaluation markedly germane to this cohort [1,19]. However, because differential toxicities of AA and ENZ and their interactions with patient co-morbidities have not been fully elucidated, there is little guidance on how to choose these drugs based on pre-existing conditions.

This scoping review aims to describe the differential outcomes, ADEs, and systemic costs of AA and ENZ for metastatic castration-resistant PCa (mCRPC) in a real-world population. The findings of this review may guide future clinical studies and ultimately facilitate tailored treatment based on the health condition of the patient.

## 2. Materials and Methods

The scoping review followed the framework outlined by the Joanna Briggs Institute Manual for Evidence Synthesis (JBIMES), incorporating protocols established by Arksey and O’Malley along with revisions from Levac et al. and Peters et al. [20,21,22,23,24]. The study included the following six steps: defining the research question; identifying relevant studies; study selection; charting the data; collating, summarizing, and reporting the results; and consultation. Findings were reported according to the Preferred Reporting Items for Systematic Reviews and Meta-Analyses (PRISMA) guidelines, utilizing the extension for scoping reviews (PRISMA-ScR) [25] and the PRISMA-ScR checklist. The study protocol was registered prior to commencement at Figshare, available at https://doi.org/10.6084/m9.figshare.19149227.v3 (accessed on 28 June 2022) [26].

### 2.1. Study Scope

The study was intended to evaluate patient outcomes following AA and ENZ utilizing data on drug-associated toxicities, mortality, hospitalizations, HRU, costs, patient adherence, and patient comorbidities. The definition of outcomes was purposefully left broad, as a variety of disease-specific, patient-reported, and systemic outcomes are important to clinical decision making. The review focused on real-world cohort studies to create an analysis applicable to the general population. Studies with rigorous patient exclusion criteria, such as clinical trials, were excluded.

### 2.2. Search Strategy

The search strategy for this review was informed by prior PCa therapy research, as well as recommendations by Tawfik et al. to adapt searches to the database being utilized [27]. An experienced search librarian was consulted. Searches of PubMed, Cochrane Library, CINAHL, and Scopus were conducted using the following keywords: “prostate cancer”, “prostatic neoplasms”, “abiraterone acetate”, “enzalutamide”, “toxicities”, outcomes”, and associated MeSH terms. These terms were combined with the Boolean operators “AND” and “OR”. The initial search was performed in PubMed using relevant MeSH terms, and similar searches were used for Cochrane Library and CINAHL, which also utilize MeSH terms. Only keywords were used for Scopus. The final search strings are provided in Table 1.

The search was restricted to full-length peer-reviewed English publications through 31 January 2022. Free-standing abstracts, opinion pieces, reviews, and letters to the editor were excluded. Due to their abbreviated format, abstracts do not present all relevant data, while editorials are opinion-based.

This review captured studies that compared outcomes and toxicities of AA and ENZ, focusing on retrospective institutional or population-based cohort studies. Studies investigating only one of the treatments were also excluded. If further information was required, the respective authors of the publications were contacted. The reference lists of all included articles were also searched for additional studies.

### 2.3. Data Charting and Extraction Process

Endnote 20 (Clarivate, Philadelphia, PA, USA) was employed for imported reference management and duplication removal. Studies identified by the above search strategy to satisfy the initial inclusion criteria were considered for title, abstract, and keyword screening by three independent reviewers. Articles satisfying initial screening underwent full-text screening by three independent reviewers. Lack of unanimity regarding determination of study eligibility at each stage was resolved through discussion with a senior member of the research team.

Three members of the research team (YBS, JB, and SM) independently conducted data extraction. From each article, the following information was extracted: author, year of publication, title, drug treatment regimen, study type/design, study population, primary objective(s), outcome(s), and primary conclusions (Table 2). 

### 2.4. Synthesis, Reporting of Results, and Consultation

Given the broad scope of the research question and wide-ranging definition of patient outcomes and limited availability of published real-world cohort studies, a narrative synthesis and reporting of results was chosen. Extracted outcomes were wide-ranging in context and form, making a systematic review and meta-analysis suboptimal. Expert clinicians (WKK and SJF), who serve as the primary stakeholders in the determinations of this study, were consulted to inform data interpretation and subsequent discussion of clinical implications.

## 3. Results

### 3.1. Selection of Sources

**Figure 1:**
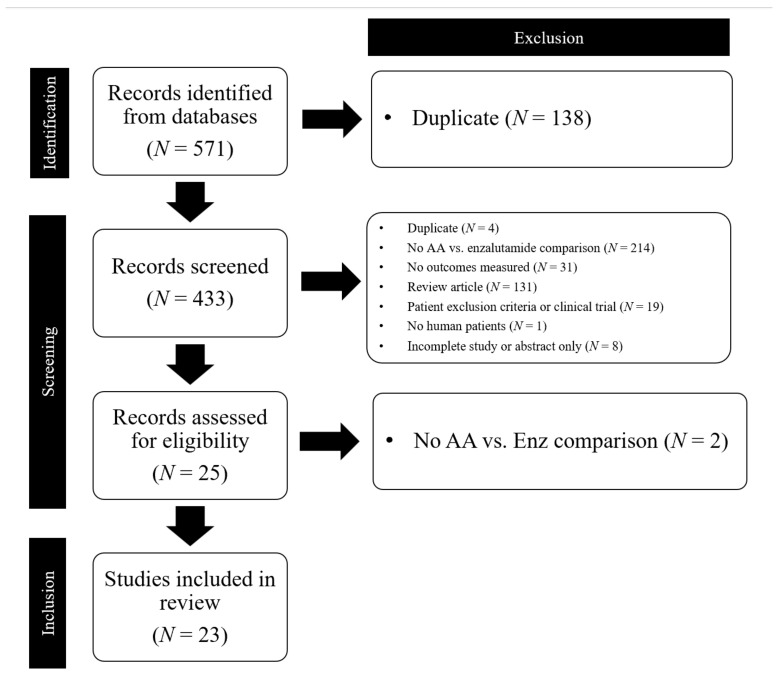
PRISMA Flow Chart of studies included in the scoping review. AA = abiraterone acetate; Enz = enzalutamide.

The systematized literature search retrieved 571 articles, of which 118 duplicates were removed by computer software and an additional 20 duplicates were eliminated manually. The titles, abstracts, and keywords of the remaining 433 publications were screened, and 25 records were ultimately identified for full-text review. Following full-text screening, 2 articles were excluded as they did not directly compare AA and ENZ. Hence, 23 studies were included in this scoping review. The most common reasons for exclusion were a lack of direct AA versus ENZ comparison, a review article format, and a lack of measured outcomes (Figure 1).

### 3.2. Characteristics of Included Sources

The studies’ characteristics are summarized in Table 2. The studies ranged in time of publication from 2014 to 2022, and were all observational, prospective cohort, retrospective, or population-based studies. Included studies measured a variety of metrics from disease-specific outcomes including cancer progression, response, and survival; treatment-associated toxicity; patient adherence; treatment duration; dose reduction; HRU; and healthcare costs. 

### 3.3. Comparison of Disease Progression and Survival

Better mCRPC response to ENZ was widely observed, although concomitant survival benefit was only occasionally found. Jarimba et al. demonstrated higher response rates with ENZ versus AA (77.1% vs. 58.1%, *p* = 0.016) and showed that positive response independently reduced risk of both biochemical progression (OR: 0.248, *p* = 0.017) and death (OR: 0.302, *p* = 0.038), but still found no significant difference in all-cause time to death (37.5 months ENZ vs. 26 months AA, *p* = 0.277) [2]. Higher PSA response with ENZ (61.6% vs. 43.8%, *p* < 0.004) and greater time to progression (HR 0.66; 95% CI 0.50–0.88, *p* < 0.01) were recorded by Soleimani et al. [28]. Similarly, biochemical response was attained much more quickly with ENZ (7 vs. 15.5 weeks) in the Caffo et al. study, who also found improved biochemical PFS, with over 50% PSA reduction in 23/31 ENZ vs. 8/26 AA patients at one month after treatment [29]. Miyake et al. demonstrated median biochemical PFS of 11.6 months in ENZ versus 9.0 months in AA (*p* = 0.014) and PSA response in 70.7% of ENZ versus 53.1% of AA patients, all of which were inferior to randomized clinical trials. PSA progression occurred in 30.5% and 54.9% of ENZ and AA patients [30]. Pilon et al. found that AA had a 19.0% reduction in risk of death compared to placebo; the study compared this rate to previous literature which found much higher risk reductions with ENZ (23–37%), though statistical comparisons could not be made [31].

Three reviewed studies found a significant overall survival (OS) difference between ENZ and AA, all in favor of ENZ. Tagawa et al. reported that ENZ patients had a 16% lower risk of death (adjusted HR = 0.84; 95% CI, 0.76–0.84; *p* = 0.0012), with increased median OS (29.63 months vs. 25.87 months) [32]. Scailteux et al. suggested a 10% improved OS with ENZ versus AA (34.2 m vs. 31.7 m, HR 0.90, 95% CI 0.85–0.96) [33]. Demirci et al. found longer radiographic progression-free survival (rPFS) and OS with ENZ (15 m vs. 7 m, *p* < 0.001 and 29 m vs. 16 m, *p* = 0.027), alongside more frequent PSA decline greater than 50% (*p* = 0.020) [34].

Four other studies found the two therapies more comparable, although, notably, no studies reported the superiority of AA. A mean OS of 18.9 ± 1.5 months with no significant differences between ENZ (24 months) and AA (15 months) was found by Al-Ali et al. [35]. Similarly, no significant difference in rPFS was observed by Banna et al. (12.8 m ENZ vs. 17.4 m AA, *p* = 0.30), nor Chang et al. (9.5 m ENZ vs. 7.3 m AA, *p* = 0.766) [36,37]. ENZ patients had a better biochemical response, PSA response preservation, and PSA decline, though none of these measures were statistically significant [37]. Chowdhury et al. found that time to progression was comparable and median OS was identical for both groups (27.1 m) [38].

Age, co-morbidities, and disease stage played an important role when included in multivariate models. Importantly, Miyake’s study reported that AA was more commonly selected for patients with unfavorable characteristics [30]. Baseline inferior health of AA patients was supported by Banna et al. (*p* = 0.04) and Demirci et al. (*p* = 0.016) although refuted by Chowdhury et al., who identified de novo metastases at diagnosis in 35.0% of AA and 42.3% of ENZ patients [36]. Behl also found that AA patients were typically older (73.8 years vs. 72.8 years, *p* = 0.02).

Generally, most studies indicated superior response and sustained PFS with ENZ, with three also reporting increased OS. The remaining studies cited low sample size as the primary reason for a lack of significance, but still noted that ENZ seemed to improve disease-specific outcomes. Four studies did note a trend towards AA prescription for patients with unfavorable disease factors, which confounds signals of ENZ superiority.

### 3.4. Comparison of Drug-Associated Toxicities

We reviewed included articles to uncover trends in ADEs and differences in common types of toxicities between AA and ENZ, along with associations with patient co-morbidities. Jarimba et al. reported ADEs in 16.1% of AA and 11.4% of ENZ patients, while Chowdhury et al. found that 7.1% of AA and 13.5% of ENZ patients discontinued treatment due to toxicity, and death during the treatment period was seen in 7.2% of AA and 11.1% of ENZ patients [2,38].

We first reviewed the association of inherent patient characteristics with treatment exposure and subsequent toxicity. Importantly, older age was not reported to affect treatment exposure for either AA or ENZ, an important negative finding which confirms post hoc analyses of clinical trials [39]. Similarly, body mass index or liver function did not influence treatment exposure, although estimated glomerular filtration rate did influence exposure to both AA and ENZ (*p* = 0.002 and *p* < 0.001) [39].

Cardiovascular toxicities following AA were noteworthy; such patterns were less notable in ENZ patients. Lu-Yao et al. reported that AA was associated with higher post-treatment hospitalization rates among those with pre-existing CVD. In particular, patients with ≥3 CVD conditions had a 41% lower post-treatment hospitalization risk when treated with ENZ compared to AA after adjustment for potential confounding variables (IRR 0.59; 95% CI 0.44–0.79) [18]. George et al. demonstrated CVD toxicities for both AA (HR 1.23; 95% CI 1.05–1.45) and ENZ (HR 1.10; 95% CI 1.00–1.21) compared to the control, although the risk was higher with AA; these findings were corroborated by several reviewed studies [40]. Stratification of specific cardiovascular toxicities demonstrated that AA was associated with hypertension, cardiac toxicity, fluid retention, and hypokalemia, while ENZ was associated with hypertension alone.

Central nervous system (CNS) toxicities including amnesia, cognitive disorders, confusion, and memory impairment; fatigue; and hot flashes were more common in patients treated with ENZ [2,15,41]. Specifically, one study identified more frequent fatigue in ENZ versus AA (18% vs. 4%, *p* = 0.04) [42], and another demonstrated that fatigue was the most common reason for ENZ dose reductions (30.4%) or discontinuation (5.6%) [28]. Similarly, a third study found that fatigue was more commonly observed in ENZ (32.3% vs. 19.4%). Liver toxicity was very common in AA patients, affecting 11.5% of this cohort, compared to only 5.4% of ENZ patients [30]. Another study had to withdraw two (3.1%) AA and two (15.4%) ENZ patients for Grade 3/4 liver function impairment and Grade 3/4 fatigue, respectively [37].

### 3.5. Comparison of Treatment Adherence, Dose Reduction, and Dose Modification

We sought to elucidate therapy-related factors which may impact differential adherence rates between AA and ENZ, as adherence can greatly impact outcomes in the real-world setting. The World Health Organization has reported categories which impact patient adherence, including therapy-related toxicities and complexity of treatment, among other factors such as patient beliefs and mental health, disease-related factors and co-morbidities, and the clinician–patient relationship.

Generally, studies found satisfactory adherence rates for both AA and ENZ, reporting medication possession ratios of 90% and 85% and non-adherence rates of 4.8% and 6.2% for AA and ENZ, with no significant differences between the two [35,36,43], Importantly, differential adherence rates did not affect OS, which was predominantly affected by pre-existing co-morbidities [36,44].

Behl et al. included a Kaplan–Meier curve demonstrating that lower dose reduction risk in AA became most evident at 3 m follow-up, with the difference becoming more pronounced at greater follow-up periods [45]. Similarly, they reported longer exposure to treatment with AA (7.5 ± 6.1 months vs. 6.3 ± 5.9 months; *p* < 0.0001). They proposed that high rates of fatigue with ENZ may explain lower adherence. Shore et al. similarly demonstrated more dose reductions with ENZ (16% vs. 6%), and attributed this to ADEs [41], a finding supported by Soleimani et al. (44.8% vs. 22.9%, *p* < 0.001). Fewer AA versus ENZ patients required dose reduction for reasons besides disease progression (28.8% vs. 40.8%, *p* = 0.04), resulting in lower dose exposures, although this did not harm outcomes. Interestingly, time to progression was higher in ENZ patients requiring dose reduction [28].

Freedland et al. conversely found that dose reductions were not more common or intense in ENZ versus AA. Moreover, dose reductions were seen in 64.4% of all study patients and were associated with 8.8% increased risk of biochemical progression. Dose reductions are relevant given that the combination of ENZ with a CYP2C8 inhibitor can increase ENZ levels by 2.2-fold, thereby potentially requiring a lower dose [46].

Finally, Pilon et al. found treatment duration was significantly higher with AA (18.3 m vs. 14.2 m, *p* < 0.001), and AA patients experienced fewer discontinuations (HR = 0.73; *p* = 0.004) across a 24 m span [31]. Two studies actually reported a longer median treatment duration in ENZ versus AA (19.7 vs. 8.8 m and 9.93 m vs. 8.47 m, *p* = 0.0008) [32,47].

**Table 2:** Selected study characteristics.

Authors	Study Design	Population Characteristics	Outcomes Reported
Al-Ali, B. et al. (2018) [35]	Retrospective population-based database	CRPC patients (*N* = 457), mean age 74.4 y, AA *N* = 195, ENZ *N* = 139	OS, MPR, treatment duration, length of hospital stay
Banna, G. et al. (2020) [36]	Observational prospective cohort	mCRPC patients (*N* = 58), median age 76 y, AA *N* = 22, ENZ *N* = 36	Cancer response, OS, radiographic PFS, adherence
Behl, A. et al. (2017) [45]	Retrospective population-based database	mCRPC patients, AA *N* = 2591, ENZ *N* = 807	OS, MPR, dose reduction
Caffo, O. et al. (2014) [29]	Observational prospective cohort	Progressive CRPC patients, AA *N* = 26, ENZ *N* = 31	Cancer response, cancer progression, toxicities
Chang, L. et al. (2019) [37]	Retrospective single-institutional cohort	mCRPC patients with prior docetaxel treatment, AA *N* = 64, ENZ *N* = 13	Cancer response, OS, PFS, toxicities
Chowdhury, S. et al. (2020) [38]	Retrospective population-based database	mCRPC patients, AA *N* = 754, ENZ *N* = 227	Time to progression, OS, treatment duration
Cindolo, L. et al. (2019) [44]	Retrospective population-based database	mCRPC patients, AA *N* = 109, ENZ *N* = 14	Drug persistence, adherence
Crombag, M. et al. (2019) [39]	Retrospective single-institutional cohort	CRPC patients, AA *N* = 71, ENZ *N* = 64	Drug exposure by co-morbidity
Demirci, A. et al. (2021) [34]	Retrospective multi-institutional cohort	mCRPC patients (*N* = 250)	Treatment response, radiographic PFS, OS
Freedland, S. et al. (2021) [46]	Retrospective population-based database	mCRPC patients (*N* = 6069)	Dose reduction
George, G. et al. (2021) [40]	Retrospective population-based database	CRPC patients, AA *N* = 1310, ENZ *N* = 3579	Toxicities (cardiovascular)
Hu, J. et al. (2021) [15]	Retrospective population-based database	mCRPC patients (*N* = 2183), AA *N* = 1773, ENZ *N* = 410	Hospitalizations, toxicities (cardiovascular)
Jarimba, R. et al. (2021) [2]	Retrospective single-institutional cohort	mCRPC patients (*N* = 91), AA *N* = 56, ENZ *N* = 35	Treatment response, PFS, toxicities
Lu-Yao, G. et al. (2019) [18]	Retrospective population-based database	CRPC patients, AA *N* = 2845, ENZ *N* = 1031	Mortality, hospitalizations, toxicities (cardiovascular)
Miyake, H. et al. (2017) [30]	Retrospective single-institutional cohort	mCRPC patients (*N* = 280), AA *N* = 113, ENZ *N* = 167	Treatment response, cancer progression, toxicities
Pilon, D. et al. (2017) [31]	Retrospective population-based database	mCRPC patients, *N* = 3398, AA *N* = 2591, ENZ *N* = 807	Treatment discontinuation, treatment duration
Ramaswamy, K. et al. (2020) [48]	Retrospective population-based database	mCRPC patients (*N* = 3174), AA *N* = 1945, ENZ *N* = 1229	HRU, costs
Salem, S. et al. (2017) [42]	Retrospective single-institutional cohort	mCRPC patients (*N* = 189), AA *N* = 76, ENZ *N* = 113	Treatment duration, dose reduction, toxicities
Scailteux, L. et al. (2020) [33]	Retrospective population-based database	CRPC patients (*N* = 10,308), AA *N* = 6585, ENZ *N* = 3723	OS
Schultz, N. et al. (2018) [47]	Retrospective population-based database	mCRPC patients, AA *N* = 2310, ENZ *N* = 920	Treatment duration, hospitalizations, HRU, costs
Shore, N. et al. (2019) [41]	Prospective Phase IV surveillance study	mCRPC patients with exclusion of those with prior chemotherapy, seizure disorder, dementia, or substance abuse, *N* = 92, AA *N* = 46, ENZ *N* = 46	Dose reduction, toxicities
Soleimani, M. et al. (2021) [28]	Retrospective single-institutional cohort	mCRPC patients aged ≥ 80 years (*N* = 278), AA *N* = 153, ENZ *N* = 125	Treatment response, cancer progression, dose reduction
Tagawa, S. et al. (2021) [32]	Retrospective population-based database	mCRPC patients, AA *N* = 1229, ENZ *N* = 1945	OS, treatment duration, toxicities

Note: AA = abiraterone acetate; Enz = enzalutamide; mCRPC = metastatic castration resistant prostate cancer; MPR = medication possession ratio; OS = overall survival; PFS = progression free survival; HRU = healthcare resource utilization.

### 3.6. Comparison of Resource Utilization, Hospitalization, and Cost

For cohorts where disease-specific outcomes, co-morbidity interactions, or toxicity sensitivities are not clearly different between AA and ENZ, systemic factors such as total cost or HRU might inform treatment choice. These factors can impact quality of life and public health, but are not commonly studied as relevant outcomes, and, hence, only three studies primarily reported such data.

Al-Ali et al. indicated the importance of HRU as an outcome of relevance by remarking that only 9.4% of study patients were never hospitalized, and patients spent an average of 13% of their remaining life span in the hospital. Hospital stays were generally longer with AA versus ENZ (39.4 ± 36.8 days vs. 26.3 ± 25.8 days) [35]. Increased costs were largely linked to risk of hospitalizations or healthcare visits and length of hospital stays. Of note, these data were from Austria, and exact costs may differ across countries, particularly in the United States, although relative comparisons likely remain consistent.

Ramaswamy et al. found fewer all-cause outpatient or pharmacy visits and costs per patient per month (PPPM) for ENZ patients in the United States. The ENZ cohort experienced fewer all-cause inpatient (2.51 vs. 2.86, *p* < 0.0001) and PCa-related outpatient visits (0.86 vs. 1.03, *p* < 0.0001). This corresponded with lower all-cause (USD 2588 vs. USD 3115, *p* < 0.0001) and PCa-related (USD 1356 vs. USD 1775, *p* < 0.0001) outpatient costs PPPM. Total medical plus pharmacy costs were also lower with ENZ (USD 8085 vs. USD 9092, *p* = 0.0002 and USD 6321 vs. USD 7280, *p* < 0.0001). Overall, emergency room, cancer-related visit, cancer-related pharmacy, and all-cause costs were all lower with ENZ, and a yearly cost benefit of USD 12,000 was identified [48]. The authors also noted that differences in HRU and cost were significantly larger than those expected from clinical trials, attributing the discrepancy to higher HRU by patients who were ineligible for trials.

Schultz et al. supported these findings. ENZ patients reported fewer all-cause inpatient admissions (IRR 0.87; 95% CI 0.76–0.99), days of hospitalization (IRR 0.84; 95% CI 0.70–1.02), and outpatient visits (IRR 0.94; 95% CI 0.90–0.98), alongside fewer PCa-related outpatient visits (IRR 0.92; 95% CI 0.87–0.96) versus AA. Furthermore, within 3 m of treatment initiation, ENZ patients visited the emergency department for PCa-related concerns at 28% lower rates (adj OR 0.72; 95% CI 0.53-0.98) and were admitted as inpatients at 24% lower rates (adj OR 0.76; 95% CI 0.57-1.02). Although monthly pharmacy costs were USD 545 higher for ENZ (*p* < 0.001), this drug expense was negated by lower total healthcare costs compared to AA. Upon adjusted differences of USD 485 for total pharmacy costs (*p* < 0.001) and USD 834 for index drug costs (*p* < 0.001), this resulted in adjusted cost savings of USD 28 (*p* = 0.009) for emergency department visits and USD 122 (*p* = 0.024) for inpatient admissions with ENZ [47].

Focusing on cardiovascular risk, two additional studies further clarified differences in HRU. AA patients had increased risk of all-cause (HR 1.26; 95% CI 1.04–1.53; *p* = 0.019), CVD-related (HR 1.82; 95% CI 1.09–3.05, *p* = 0.022), and heart failure-related (HR 2.88; 95% CI 1.09–7.63) hospitalizations [15]. As previously mentioned, patients with three or more CVDs had a 41% lower hospitalization rate when administered ENZ versus AA (IRR = 0.59; 95% CI 0.44–0.79) [18].

## 4. Discussion

This review found notable differences in treatment outcomes following AA versus ENZ among patients with mCRPC (Table 3). In general, ENZ is associated with more favorable survival and disease control. Three studies demonstrated better survival following ENZ compared with AA [32,33,34]. Several studies showed a similar trend but did not reach statistical significance, primarily due to low sample sizes during subgroup analyses performed to exclude confounding patient factors. Patients with pre-treatment risk factors or lower baseline prognosis more likely received AA. Nonetheless, most studies agreed that ENZ patients demonstrate increased biochemical or radiographic response. Although significance is difficult to achieve given variable patient populations and small cohort sizes, it appears that ENZ is demonstrably superior in disease control [4,49].

A previous review of clinical trials which indirectly compared AA and ENZ found that OS and cancer progression were slightly better for the latter, corroborating our findings. It also reported significant superiority of ENZ for secondary measures including biochemical response, biochemical progression, and rPFS. However, this study similarly lost significance upon subgroup analysis [3]. Another review of trials supported these findings, but without direct comparison of the two drugs, potential translation to treatment choice algorithms remains limited [50].

Universally, as expected, disease-specific outcomes were inferior in included studies when compared to clinical trials. This can be attributed to patient selection and strict exclusion criteria eliminating co-morbid patients from trials [51]. One clinical trial review found an OS advantage of 4.6 m and 4.8 m for AA and ENZ [52]. Without NHTs, median OS of metastatic castration-resistant PCa is 14 m, meaning these drugs can extend survival by approximately 35% [53].

It is important to note that clinical trials certainly hold value compared to retrospective analyses, where the patient cohort is extremely heterogeneous and may include patients with PCa at different timepoints in the natural history of disease. Hence, future comparative studies with different risk sub-groups may hold utility in analyzing treatment choice for the real-world PCa population. Larger population-based studies which can control for patient heterogeneity may also robustly demonstrate a difference in efficacy and CRPC response. Despite recent approvals of these drugs in castration-sensitive PCa, this scoping review did not identify any study with such patients, indicating further study is also essential in this setting.

Interestingly, two studies reported differences in AA outcomes based on race and ethnicity, finding that African American men experienced improved PSA response and OS compared with non-Hispanic White men [54,55]. These findings warrant further analyses of the differential impacts of race and ethnicity on AA versus ENZ outcomes to determine the utility of these patient-specific traits in treatment assignment algorithms. Reduced PFS following AA was exhibited in patients with diabetes, although this has not yet been reported in ENZ [56]. Further research is necessary to clarify whether patients with pre-existing diabetes may benefit more from one treatment versus another.

### 4.1. Consideration of Treatment Toxicities

This review found significant and noteworthy differences in drug-associated toxicities between AA and ENZ. Although both drugs demonstrated largely similar rates of drug-related AEs and high-grade AEs, the specific toxicities differed. Therefore, pre-existing conditions may be considered when assigning patients to a therapy.

In the clinical trial setting, AA was associated with fluid retention, hypertension, and hypokalemia [47]. Because clinical trials often exclude patients with significant co-morbidities, we believed that compiling ADEs from various smaller studies would clarify the common toxicities that clinicians may expect and monitor in the real world. We found that while types of toxicities largely remained consistent, overall ADEs occurred more frequently outside the trial setting.

Importantly, our analysis demonstrated that age and liver function do not appear to influence treatment exposure. However, AA is associated with a higher risk of liver toxicity. In the real-world, most men with pre-existing impaired liver function should avoid AA. Drug–drug interactions were not analyzed in this review but comprise a significant source of toxicity. As previously described, ENZ exposure can increase notably with concurrent CYP2C8 inhibitors, making this an important point of future inquiry.

Although both AA and ENZ have been shown to increase major cardiovascular events requiring hospitalization [18,57], namely, AA was shown to confer significantly more cardiovascular toxicities. One meta-analysis of clinical trials reported that AA confers a 2.2-fold risk of cardiovascular toxicity (RR 2.2; 95% CI 1.60–3.27) through post-treatment hospitalizations across all CVD categories examined, although mortality differences were not observed. This risk was not seen with ENZ (RR = 1.32; 95% CI: 0.85–2.06) [18]. Elucidating interactions with CVDs is important, as two-thirds of Medicare patients treated with NHTs had pre-existing CVD conditions, and these patients experience 23–37% higher mortality [18]. Further study is needed to evaluate whether patients with several CVD conditions will have more favorable outcomes with ENZ than with AA.

The toxicities of prednisone, which is typically administered alongside AA, must also be considered, as long-term use of corticosteroids have well-elucidated detrimental effects [8]. Mineralocorticoid-related AEs, including hypertension, fluid retention, and hyperkalemia, are also associated with AA [3,5,53]. AA may be less advisable in patients with CHF, renal failure, or metabolite disturbances [18].

Conversely, our results confirmed that fatigue is a well-established and commonly observed toxicity of ENZ, alongside other CNS toxicities such as amnesia, confusion, and cognitive disorders. Patients with neurological risk factors may be less suited for ENZ treatment, and those receiving ENZ may be closely monitored, particularly if they have a known seizure disorder, brain metastases, or brain injury.

Many of these findings have been demonstrated in clinical trials, although a direct comparison between AA and ENZ has not been made. As expected, fatigue with ENZ was more common in our review of real-world studies than in trials [58].

### 4.2. Differences in Systemic Healthcare Quality Metrics

Our study unequivocally found that ENZ resulted in reduced total healthcare costs and HRU, with cost-related studies focusing on the United States and HRU-related analyses being consistent across countries. Although ENZ was more expensive in direct pharmacy costs, ENZ patients were less likely to require other healthcare services, and, hence, incurred fewer net costs. Similarly, HRU was significantly lower for ENZ patients, with significantly fewer post-treatment outpatient, inpatient, and emergency room visits. Further study is necessary to evaluate whether this financial toxicity affects accessibility and treatment outcomes for patients based on socioeconomic and geographic factors.

In terms of drug costs alone, a previous cost effectiveness analysis found that AA has a 28% lower cost than ENZ [59]. This study assumed that treatment duration and OS were shorter in AA. Even after accounting for treatment durations or recommended treatment-specific monitoring costs, AA was cheaper. However, total cost incurred is more relevant than pharmacy expenses alone, and total costs are consistently lower with ENZ.

A previous study of oral targeted therapies found that both AA and ENZ precipitated greater all-cause healthcare costs than older agents, namely docetaxel and prednisone [60]. Numerous other cost-effectiveness analyses have corroborated this finding. All-cause monthly total healthcare costs for patients receiving NHTs were estimated at approximately 15,000 (2017 USD).

Although secondary hormonal therapies may delay disease progression, they often confer significant AEs, which may increase costs and HRU while reducing quality of life. One study reported CNS ADEs in nearly 46% of ENZ patients [61]. Our review demonstrates that ENZ-associated fatigue and other ADEs impacted adherence and dose reduction rates but did not increase costs or HRU when compared to AA-associated ADEs. As discussed, because AA is administered concomitantly with prednisone, it was expected that AA would result in increased healthcare needs.

Notably, post-NHT HRU cannot be entirely attributed to drug-related adverse effects. This patient cohort experiences several complications related to mCRPC itself, including severe skeletal-related events (SREs) such as spinal cord compressions and fractures due to bony metastases. Importantly, both AA and ENZ have been shown to significantly delay SREs, although bone modulating agents are now recommended in combination with either of these antiandrogens to further reduce incidence. Nonetheless, PCa-related complications such as SREs can contribute to post-NHT HRU, and differences in their frequency between AA and ENZ should be analyzed in future comparisons [62,63].

Direct healthcare costs of castration-resistant PCa range from USD 2474 to USD 67,957 annually. These costs increase five-fold upon metastasis, and HRU increases 1.5–2.5-fold [60]. Given the significant cost and healthcare need, it is important to assign treatments which minimize healthcare burdens at the patient and systems-based levels. These measures are important systems-based considerations when determining treatment choice, particularly when disease-specific outcomes may appear equivocal and patient history may not directly exclude one of the drug options.

### 4.3. Strengths and Limitations

This study had several strengths. The review utilized a strong, transparent methodology based upon a previously registered protocol. A broad search was conducted in four major databases, and studies were evaluated independently by three reviewers. Finally, a multidisciplinary team was involved throughout the study, including expert clinicians who informed this study’s discussion of clinical implications. Nonetheless, this scoping review has various limitations, including the exclusion of abstracts and non-English articles. Given the retrospective nature of included studies, patient cohorts were heterogenous. Although individual studies performed multivariate adjustments to adjust for co-morbidities and other modulating factors, the full extent of inter-cohort differences cannot be elucidated in a scoping review, and, hence, these findings should be interpreted with caution. A further limitation includes the lack of information surrounding patient diversity, including data on patient race and ethnicity, which limits broad applicability. Finally, synthesis of results and summative statistical analysis was limited given the broad definition of outcomes and the relatively small amount of data available for each individual outcome.

## 5. Conclusions

This is the first scoping review to compare the treatment outcome of AA vs. ENZ amongst mCRPC patients. Existing retrospective and real-world studies indicate that AA and ENZ have profoundly differing effects based on patient risk factors, as AA is associated with notable cardiovascular toxicities while ENZ has neurological adverse effects and fatigue. AA demonstrates poorer systemic outcomes including HRU and cost, while it shows superior treatment adherence. Finally, ENZ offers faster and more frequent disease response, and select studies demonstrated improved survival, although these outcomes are still unclear. Real-world outcomes data with both agents were inferior to results seen in clinical trials. These differences may become increasingly relevant as many AA and ENZ users have a higher comorbidity burden than those in clinical trials and NHT indications are expanding to earlier disease stages [64]. The results of this scoping review suggest that further research comparing patient-specific and systemic measures following AA and ENZ therapy is needed. Further study may inform an evidence-based, patient-centered clinical decision making algorithm to optimize PCa patient outcomes and experiences.

## Figures and Tables

**Table 1:** Search strings utilized.

Database	Search String	Result
**PubMed**	(“Prostatic Neoplasms”[MeSH Terms] OR “Prostatic Neoplasms”[All Fields] OR “prostate cancer”[All Fields]) AND (“Abiraterone Acetate”[MeSH Terms] OR “Abiraterone Acetate”[All Fields]) AND (“enzalutamide”[All Fields] OR “Androgen signaling inhibitor”[All Fields]) AND (“Drug-Related Side Effects and Adverse Reactions”[MeSH Terms] OR “Treatment Outcome”[MeSH Terms] OR “Hospitalization”[MeSH Terms] OR “Mortality”[MeSH Terms] OR “Comorbidity”[Mesh])	169
**Scopus**	TITLE-ABS-KEY ((“Prostatic Neoplasms” OR “prostate cancer”) AND (“Abiraterone Acetate”) AND (enzalutamide OR “Androgen signaling inhibitor”) AND (“Drug-Related Side Effects and Adverse Reactions” OR “Treatment Outcome” OR hospitalization OR mortality OR comorbidity)) AND (LIMIT-TO (DOCTYPE, “ar”) OR LIMIT-TO (DOCTYPE, “re”) OR LIMIT-TO (DOCTYPE, “no”)) AND (LIMIT-TO (LANGUAGE, “English”))	399
**CINAHL**	((MH “Prostatic Neoplasms+”) OR “Prostatic Neoplasms” OR “prostate cancer”) AND ((MH “Abiraterone Acetate+”) OR “Abiraterone Acetate”) AND (enzalutamide OR “Androgen signaling inhibitor”) AND ((MH “Drug-Related Side Effects and Adverse Reactions+”) OR (MH “Treatment Outcome+”) OR (MH Hospitalization+) OR (MH Mortality+) OR (MH Comorbidity+))	3
**Cochrane Reviews**	([mh “Prostatic Neoplasms”] OR “Prostatic Neoplasms” OR “prostate cancer”) AND ([mh “Abiraterone Acetate”] OR “Abiraterone Acetate”) AND (enzalutamide OR “Androgen signaling inhibitor”) AND ([mh “Drug-Related Side Effects and Adverse Reactions”] OR [mh “Treatment Outcome”] OR [mh Hospitalization] OR [mh Mortality] OR [mh Comorbidity])	0

**Table 3:** Summary of findings.

Outcomes	AA	ENZ
**Disease progression and survival**		
Higher biochemical response rate [28,29,30,31]		X
Improved biochemical progression-free survival [29,30,31]		X
Improved OS or rPFS [33,34,35]		X
*Comparable OS or rPFS* [36,37,38,39]	*Equal*	
Baseline inferior patient health [31,35,37,46]	X	
**Drug-associated toxicities**		
Higher cardiovascular toxicities [15,18,41]	X	
Higher CNS toxicities [15,28,42]		X
Increased fatigue [15,28,29,38,42,43]		X
Increased hepatotoxicity [31,38]	X	
**Treatment adherence, dose reduction, and dose modification**		
Adherence [36,37,44]	*Equal*	
Increased dose reduction [32,42,46]		X
**HRU, hospitalization, and cost**		
Increased length of hospital stay [36]	X	
Increased all-cause cost [49]	X	
Increased HRU, visits, or admissions [48,49]	X	
Increased pharmacy costs [48]		X

Note: AA = abiraterone acetate; ENZ = enzalutamide.
